# Knock-down of methyl CpG-binding protein 2 (MeCP2) causes alterations in cell proliferation and nuclear lamins expression in mammalian cells

**DOI:** 10.1186/1471-2121-13-19

**Published:** 2012-07-11

**Authors:** Federica Babbio, Ilaria Castiglioni, Chiara Cassina, Marzia Bruna Gariboldi, Christian Pistore, Elena Magnani, Gianfranco Badaracco, Elena Monti, Ian Marc Bonapace

**Affiliations:** 1 Department of Theoretical and Applied Sciences, Insubria University, via A. da Giussano 10, Busto Arsizio, 21052, Italy; 2 Department of Biotechnologies and Life Sciences, Insubria University, via H. J. Dunant 3, Varese 21100, Italy

**Keywords:** Methyl CpG-binding protein 2 (MeCP2), Cell proliferation, Nuclear lamins

## Abstract

**Background:**

MeCP2 (CpG-binding protein 2) is a nuclear multifunctional protein involved in several cellular processes, like large-scale chromatin reorganization and architecture, and transcriptional regulation. In recent years, a non-neuronal role for MeCP2 has emerged in cell growth and proliferation. Mutations in the MeCP2 gene have been reported to determine growth disadvantages in cultured lymphocyte cells, and its functional ablation suppresses cell growth in glial cells and proliferation in mesenchymal stem cells and prostate cancer cells. MeCP2 interacts with lamin B receptor (LBR) and with Heterochromatin Protein 1 (HP1) at the nuclear envelope (NE), suggesting that it could be part of complexes involved in attracting heterochromatin at the nuclear periphery and in mediating gene silencing. The nuclear lamins, major components of the lamina, have a role in maintaining NE integrity, in orchestrating mitosis, in DNA replication and transcription, in regulation of mitosis and apoptosis and in providing anchoring sites for chromatin domains.

In this work, we inferred that MeCP2 might have a role in nuclear envelope stability, thereby affecting the proliferation pattern of highly proliferating systems.

**Results:**

By performing knock-down (KD) of MeCP2 in normal murine (NIH-3 T3) and in human prostate transformed cells (PC-3 and LNCaP), we observed a strong proliferation decrease and a defect in the cell cycle progression, with accumulation of cells in S/G_2_M, without triggering a strong apoptotic and senescent phenotype. In these cells, KD of MeCP2 evidenced a considerable decrease of the levels of lamin A, lamin C, lamin B1 and LBR proteins. Moreover, by confocal analysis we confirmed the reduction of lamin A levels, but we also observed an alteration in the shape of the nuclear lamina and an irregular nuclear rim.

**Conclusions:**

Our results that indicate reduced levels of NE components, are consistent with a hypothesis that the deficiency of MeCP2 might cause the lack of a key “bridge” function that links the peripheral heterochromatin to the NE, thereby causing an incorrect assembly of the NE itself, together with a decreased cell proliferation and viability.

## Background

MeCP2 (Methyl CpG-binding protein 2) is widely studied in neuronal systems [1] since it acquired biomedical importance with the discovery that mutations in its gene, located at Xq28 in human, determined a profound neurodevelopmental autism spectrum disorder, the Rett syndrome (RTT) [2].

In the last years, a non-neuronal role for MeCP2 has emerged in myofibroblast transdifferentiation and fibrosis [3,4], lung development [5] and cells growth. It has been reported that MeCP2 mutation determines growth disadvantage in cultured lymphocyte cells [6] and that its functional ablation suppresses cell growth of glial cells [7] and proliferation in mesenchymal stem cells [8] and prostate cancer cells [9,10].

Besides, MeCP2 plays an important role in chromatin remodelling [11] by binding to several proteins involved in large-scale chromatin reorganization and architecture [12,13]. MeCP2 and Heterochromatin Protein 1 (HP1) [14], have been shown to concentrate in the pericentromeric heterochromatin, where they play a key structural role and are involved in the control of gene expression [15,16].

Polioudaki and co-workers [17] have shown that HP1 interacts with the nuclear envelope (NE) in an acetylation-dependent manner, forming a quaternary complex with the inner nuclear membrane protein LBR (lamin B-binding receptor) and a sub-set of core histones (H3/H4), which mediate their binding. Moreover, recent findings indicate that MeCP2 interacts with LBR [18,19], suggesting that MeCP2, HP1 and LBR could be part of a complex involved in functional structures at the nuclear periphery.

The NE is composed of an outer nuclear membrane (ONM), an inner nuclear membrane (INM), nuclear pore complexes (NPCs), and nuclear lamina. The major components of the lamina are intermediate filament-like proteins, the nuclear lamins. Most adult mammalian somatic cells contain three major lamins (A, B1 and C), as well as several minor lamins (B2 and AΔ10). Separate genes encode lamins B1 and B2, whereas all of the A-type lamins (lamin A and C) are encoded by a single gene and arise through alternative splicing of a common transcript [20,21]. Given their role in maintaining NE integrity, in orchestrating mitosis, in DNA replication and transcription, in regulation of mitosis and apoptosis and in providing anchoring sites for chromatin domains [22-24], lamins and lamin-associted proteins represent essential and fundamental components for nuclear structure and nuclear dynamics. Furthermore, cumulative data suggest that NE proteins effect transcriptional silencing by recruiting chromatin with specific epigenetic marks and silencing factors able to add new epigenetic modifications to chromatin sequestered at the nuclear periphery [25].

Mutations in the *LMNA* gene cause a variety of diseases, from muscular dystrophy and lipodystrophy to systemic diseases such as premature aging syndromes [26]. Many data, moreover, support the idea that down regulation, loss and/or specific mutations in lamins cause abnormal nuclear shape [27,28], changes in heterochromatin localization at the nuclear periphery, global chromatin reorganization, possibly specific changes in the positions of genes and give rise to various conditions termed laminopathies [29].

In this work, we inferred that MeCP2 might have a role in nuclear envelope stability, thereby affecting the proliferation pattern of highly proliferating systems. Experiments were conducted to verify such hypothesis.

## Results

### Functional ablation of MeCP2 affects cells growth and alters cycle progression

**Figure 1:**
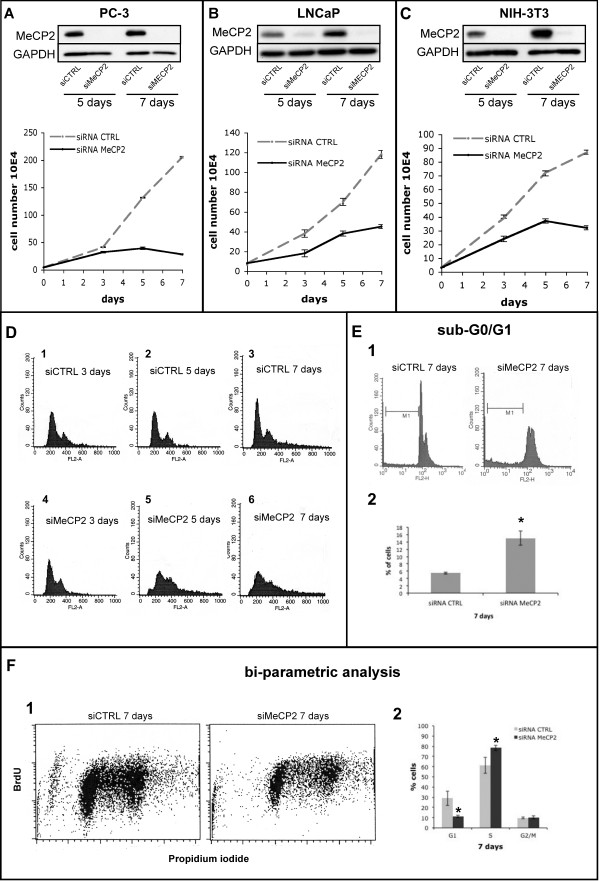
**MeCP2 ablation causes a defect in cell proliferation with a delay in cell-cycle progression.** (**A**) PC-3, (**B**) LNCaP and (**C**) NIH-3 T3 cells were transfected with siRNA MeCP2 or non-targeting siRNA CTRL oligos; MeCP2 ablation was checked at 5 and 7 days after transfection and PC-3, LNCaP and NIH-3 T3 cells, and cell proliferation was analysed (data are mean ± SD bars calculated from three independent experiments). (**D**) A representative FACS analysis of PC-3 treated with control siRNA (siRNA CTRL: plots 1, 2 and 3) or with siRNA against MeCP2 (siRNA MeCP2: plots 4, 5 and 6) that shows a delay in cell-cycle progression. (**E**) After 7 days of MeCP2 silencing was observed an enrichment of cells in sub-G_0_/G_1_ phase (M1 bar), a representative FACS analysis is shown in E1. The synthesis of three independent FACS experiments after 7 days of MeCP2 silencing is represented in bar chart E2 (statistics were performed using Student’s test. *: significant difference between siRNA CTRL and siRNA MeCP2 PC-3 cells, P < 0.005). (**F**) A representative bi-parametric BrdU/PI FACS analysis of PC-3 cells after 7 days of MeCP2 silencing is shown in F1; the synthesis of three independent bi-parametric BrdU/PI FACS analysis of PC-3 cells after 7 days of MeCP2 silencing is represented in bar chart F2 (statistics were performed using ANOVA. *: significant difference in cell cycle phases between siRNA CTRL and siRNA MeCP2 PC-3 cells, P < 0.05).

To investigate a possible role in cell cycle progression, we performed knock-down (KD) of MeCP2 by siRNA in normal murine (NIH-3 T3) and transformed human prostate cells (PC-3 and LNCaP). As shown in Figure 1, we observed a strong decrease in cell proliferation in MeCP2 depleted PC-3, LNCaP and NIH-3 T3 cells. While control cells displayed a typical exponential growth, MeCP2 KD in PC-3 cells caused a strong alteration of the growth rate and cell number. After seven days of siRNA MeCP2 treatment PC-3 cells reached only 13%ca of control (Figure 1A) indicating that the absence of MeCP2 might determine alteration in cell cycle progression. Similar results, with a 60%ca cell growth reduction in silenced MeCP2 cells have been obtained with LNCaP and mouse embryo fibroblasts (NIH-3 T3) (Figure 1B and 1C, respectively). These data are in agreement with previous published results [9,10]. 

To further investigate possible defects during the cell cycle, we performed FACS analysis of the MeCP2-ablated PC-3 and control cells (at 3, 5 and 7 days after the first transfection). Flow cytometry results underline an alteration in the cell cycle progression of MeCP2-depleted cells, with a reduction of the number of cells in the G_1_-phase and a progressive increase of cells in sub-G_0_/G_1_ (hypodiploid picks observed) and S- or G_2_M-phases beginning at the 5^th^ day of silencing, compare in Figure 1: D1-D4; D2-D5; D3-D6.

To better evaluate these cell cycle alterations in 7day siMeCP2 PC-3 cells, we detected the cell number in sub-G_0_/G_1_ by FACS analysis and the cell number in S-G_2_/M phases by bi-parametric FACS analysis (BrdU/PI incorporation). We have estimated the sub-G_0_/G_1_ cell population as non-cycling cells mentioned below the bar M1 (Figure 1 E1), and the quantitative analysis showed an increase of about 10-15% of these cells (Figure 1 E2). The results obtained after bi-parametric analyses (a representative FACS in Figure 1 F1) highlighted an increase of about 20% of cells in S-phase and a decrease of 15%ca in G_1_-phase as shown in Figure 1 F2.

Altogether, these results indicate that the MeCP2 depletion produces a consistent reduction in cell proliferation together with a defect in the cell cycle progression with an accumulation of cells in sub-G_0_/G_1_-, a decrease in G_1_-, and an increase in S-phases.

### MeCP2 silencing does not induce a severe apoptotic and/or senescence effects

**Figure 2:**
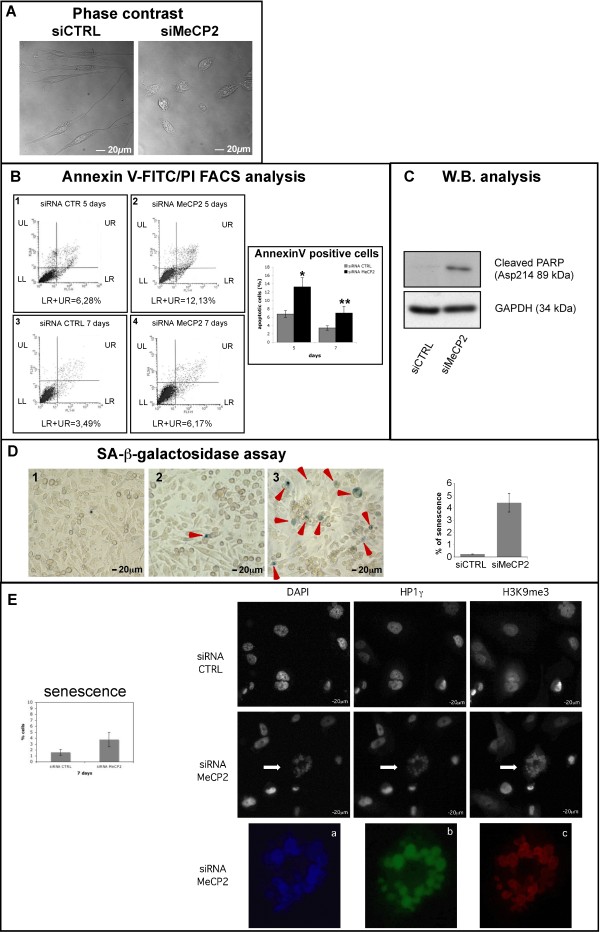
**MeCP2 silencing determines PC-3 cells suffering without triggering a severe apoptotic and senescence effects.** (**A**) Fixed PC-3 cells, transfected with siRNA CTRL or siRNA MeCP2 oligos were analyzed by confocal microscopy. Phase contrast representative pictures are shown. (**B**)Annexin V-FITC/PI FACS analysis was performed in PC-3 cells after 5 and 7 days of MeCP2 RNAi. This staining reveals the amount of early apoptosis (LR = A^+^ + PI^-^), late apoptosis (UR = A^+^ + PI^+^), living cells (LL = A^-^ + PI^-^) and necrotic cells (UL = A^+^ + PI^+^); x-axis Annexin V stain signal, y-axis PI stain signal. The percentage of apoptotic cells (UR + LR) between control and siMeCP2 samples of different experiments is shown in AnnexinV positive cells bar chart (statistic were performed using ANOVA. *: significant difference between siRNA CTRL and siRNA MeCP2 PC-3 cells after 5 days of silencing, P < 0.01. **: significant difference between siRNA CTRL and siRNA MeCP2 PC-3 cells after 7 days of silencing, P < 0.001). (**C**) PC-3 cells were transfected with siRNA CTRL and siRNA MeCP2 oligos. After 7 days of transfection protein extracts were prepared and western blot against Cleaved-PARP (Asp214 89 kDa) and GAPDH, as loading control, was performed. (**D**) SA-β-galactosidase assay was performed at 7 days after the first MeCP2 silencing. Blue cells in (D1) untreated, (D2) siCTRL and (D3) siMeCP2 cells are shown. Bar chart shown the percentage of senescent PC-3 cells after 7 days of siRNA CTRL (0.23%) and siRNA MeCP2 (4.4%) calculated by the ratio between the number of senescent and non-senescent cells in different microscope fields (data are means ± SD bars). (**E**) Identification of SAHFs (senescence-associated heterochromatic foci). Fixed 7day MeCP2 silenced PC-3 cells and control cells were treated with antibodies against HP1γ (green) and H3K9me3 (red) proteins and stained with DAPI. Cells were analysed using fluorescence microscope and an enlargement of each representative senescence stain (indicated by the white arrow) is showed (box a, box b and box c). In bar chart is indicated the percentage of senescent PC-3 cells after 7 days of siRNA CTRL (1%ca) and siRNA MeCP2 (4%ca) calculated by the ratio between the number of SAHFs and total cells in different microscope fields (data are means ± SD bars).

Decrease in cell proliferation associated to MeCP2 KD, was combined with a strong cellular suffering visualized by increased cytoplasmatic granularity and inclusions, irregular nuclear shape and absence of cytoplasmatic extroversions (Figure 2A, right panel). All these morphological characteristics added to the expression of β-galactosidase, cell cycle arrest, and hypodiplod picks (observed in siMeCP2 samples, Figure 1: D5 and D6 panels) are compatible with an apoptotic [30,31], or senescent phenotype [32-35]. This prompted us to investigate whether the absence of MeCP2 would trigger these phenotypes. We carried out transient knock-down of MeCP2 for 7 days and apoptotic cell death and senescence were assessed in MeCP2-silenced cells. 

Increased apoptosis was observed in siRNA MeCP2 PC-3 cells as compared with control cells both at 5 and 7 days after the first transfection by Annexin V-FITC/propidium iodide staining (Figure 2B: compare B1-B2; B3-B4). In both conditions (siRNA CTRL and siRNA MeCP2) the percentage of apopototic cells is low, reaching a maximum of 12%ca 5 days after MeCP2 silencing with a ratio of 2:1 of apoptotic cells between siRNA MeCP2 and siRNA CTRL (Figure 2B, Annexin-positive cells bar chart). Because of the low percentage of apoptotic cells, to confirm the phenotype observed, we evaluated the presence of the 89 kDa band of PARP that appears after cleavage of Caspase-3, one of its main targets (Figure 2C) [36].

We further detected senescence after 7 days of MeCP2 silencing, as can be inferred by the presence of SA-β-galactosidase positive blue cells in Figure 2D (compare untreated cells in D1; siRNA CTRL cells in D2 and siRNA MeCP2 in D3). In parallel, we also evaluated senescence-associated heterochromatic foci (SAHFs) by immunofluorescence (IF). This method measures changes in chromatin structures by analysing the chromatin condensation (DAPI foci) that is enriched for heterochromatin protein 1 (HP1) and histone H3 trimethylated on lysine 9 (H3K9me3) [37]. In Figure 2E the white arrow, in siRNA MeCP2 PC-3 cells, indicates a senescent cell with a large morphology and SAHFs. The enlargement of each stain DAPI (Figure 2E box a), HP1γ (Figure 2E box b) and H3K9me3 (Figure 2E box c) is shown to better evaluated the morphology.

Both SA-β-galactosidase and IF assays, suggest that the senescent phenotype observed (5%ca of 7 day siMeCP2 PC-3 cells, Figure 2D and 2E) might not be the main cause of cell growth reduction.

Overall, our results indicate that MeCP2 absence increases the percentage of senescent and apoptotic cells, although these effects do not seem to account *per se* for the strong reduction of cell number that we evidenced after 7 days of siRNA MeCP2 treatment.

### MeCP2 silencing determines alteration of nuclear envelope (NE)

Lower proliferation rates, tendency to senescence and apoptosis can be associated to structural and functional alteration of the nuclear envelope (NE) [38-41]. We, therefore, hypothesized that alterations in the NE might occur following MeCP2 depletion, thereby justifying the observed variations in the cell cycle progression.

**Figure 3:**
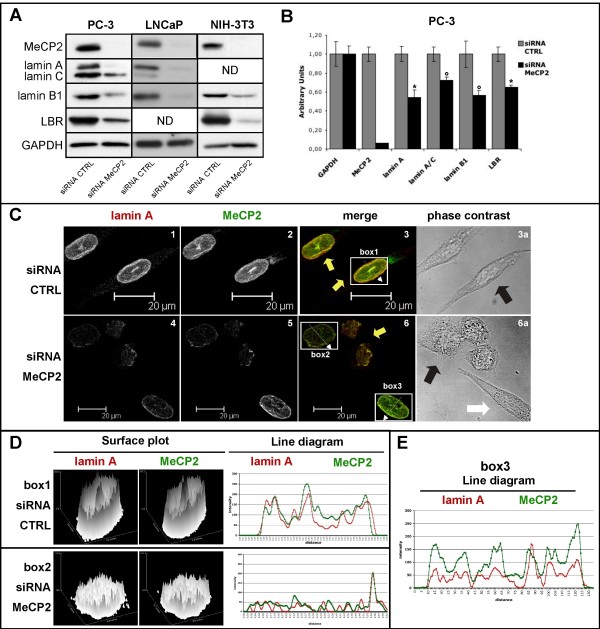
**MeCP2 functional ablation determines alteration of NE components expression.** (**A**) PC-3, LNCaP and NIH-3 T3 cells were transfected with siRNA CTRL and siRNA MeCP2 oligos. Protein extracts were prepared at 5 and 7 days after transfection. Western blot assays against MeCP2 (70 kDa), lamin A/C (69 kDa, 62 kDa respectively), lamin B1 (68 kDa), LBR (58 kDa) and GAPDH (34 kDa), as loading control, were performed. (**B**) Quantitative RT-PCR showed that 7 days of MeCP2 silencing in PC-3 cells causes alteration of mRNA levels of LMNA, lamin B1 and LBR genes. mRNAs levels were normalized with respect to GAPDH housekeeping gene and were expressed as arbitrary units (statistics were performed using Student’s test. *: significant difference between siRNA CTRL and siRNA MeCP2, P < 0.001; °, significant difference between siRNA CTRL and siRNA MeCP2, P < 0.05). (**C**) Intracellular localization of endogenous lamin A and MeCP2 proteins. Fixed 7day MeCP2 silenced PC-3 cells and control cells were treated with antibodies against MeCP2 (green) and lamin A (red) proteins and analysed by confocal microscopy. Low levels of MeCP2 protein do not alter lamin A distribution, however an irregular nuclear rim is observed (C3-C6; C3a-C6a fluorescent and phase contrast images respectively). (**D**) In the line diagram is shown the local intensity distribution (diagonal white lines through the images) of MeCP2 (green), lamin A (red) in box1 (siRNA CTRL cell) and in box2 (siRNA MeCP2) respectively. (**E**) In the line diagram is shown the local intensity distribution of MeCP2 (green) and lamin A (red) of cell in box3.

MeCP2 KD in PC-3 cells resulted in a considerable decrease in lamin A, lamin C, lamin B1 and LBR protein levels (Figure 3A). Lamin B1 was also reduced both in LNCaP and NIH-3 T3 cells (Figure 3A). Western blot experiments were confirmed by RT-qPCR analysis of the expression levels of the related genes in PC-3 cells (Figure 3B). In summary, lamin A levels were reduced up to 50%, while lamin C, lamin B1 and LBR levels were approximately reduced of 30% - 40%.

Moreover, the immunofluorescence (IF) pattern of lamin A was examined by confocal analysis in PC-3 cells after 7 days of treatment with siRNA MeCP2 (Figure 3C). These experiments confirmed the reduction of lamin A levels, without visible changes in its nuclear lamina localization. However, alterations in the shape of the nuclear lamina and an irregular nuclear rim were evident in siMeCP2 cells respect to the controls (compare in Figure 3C: panels 3 and 6, yellow arrows). These morphological abnormalities are reminiscent of features of Hutchinson-Gilford Progeria Syndrome (HGPS) [42,43], a rare premature aging disease caused by mutation in *LMNA* gene. Although there are differences between HGPS cells (LAΔ50/progerin) and MeCP2 KD cells (lamin A/C, lamin B1, LBR low levels), it is interesting to underline that, although non-overlapping, share a number of common traits [44].

We also estimated the local intensity distribution of MeCP2 and lamin A proteins; three representative cells are shown (Figure 3C: box1 of panel 3; box2 and box3 of panel 6). The siRNA CTRL cell in box1 of panel 3 of Figure 3C displays the endogenous levels of MeCP2 and lamin A, which are quantified in the top line diagram of Figure 3D. In addition, the same cell at the phase-contrast image (Figure 3C: panel 3a, black arrow) shows a normal nuclear shape. The mean fluorescence intensity levels of cell in box2 (MeCP2-siRNA treatment, Figure 3C: panel 6) show a reduction of lamin A after MeCP2 silencing (for the fluorescence intensity see Figure 3D, bottom line diagram). The same cell in the phase-contrast image (Figure 3C: panel 6a, black arrow) exhibits the characteristic nuclear shape of a suffering cell. Finally, the cell in box3 (siRNA MeCP2 treatment, Figure 3C: panel 6) represents an intermediate condition, in which a partial MeCP2 silencing is coupled with a partial lamin A depletion, without affecting nuclear shape. The line diagram of the cell in box 3 is shown in Figure 3E. This condition is also evidenced in panel 6a (white arrow) of Figure 3C, where the same cell only shows early signs of nuclear suffering.

In summary, our data indicate that the absence of MeCP2 markedly impairs nuclear envelope stability and shape by affecting the expression levels of key proteins (lamin A, lamin C, lamin B1 and LBR), suggesting that the decrease in cell proliferation observed in the absence of MeCP2 might be due to this effect. Besides, we can speculate that the apoptotic and senescence phenotypes observed in MeCP2 ablated PC-3 cells might be a consequence of nuclear envelope instability. However, we cannot exclude that the combination of all the phenotypes observed (apoptosis, senescence and alterations of NE) could cooperate on the whole to determine the strong reduction in the proliferation of PC-3 cells.

## Discussion

In this study, we have shown that the KD of MeCP2 determines a dramatic reduction in cell proliferation, combined with a strong decrease in the expression of nuclear envelope (NE) components, i.e. lamin A, C and B1 and LBR. We also detected some degree of apoptosis and senescence, as observed by other Authors [8,10], which however did not appear to be the main causes of the cell proliferation defect we encountered. It must be noted that ablation of MeCP2 produces different effects in relation to the cellular system under consideration. For example, is the previous work of Squillaro et al., [8] in human mesenchymal stem cells (MSCs) where a partial silencing of MeCP2 in MSCs induces a significant reduction of S-, along with an increase in G_1_-phase cells accompanied by a reduction of apoptosis, the triggering of senescence, a decrease in telomerase activity and the down-regulation of genes involved in maintaining of stem cell properties. In these cells senescence appeared to rely on impairment of DNA damage repair and seemed to occur through RB- and P53 related pathways. Differently, the null-p53 human AR-independent prostate cancer PC-3 cells express a distinct pattern of proteins involved in cell cycle control with a defective G_1_ checkpoint [45-47], allowing these cells to enter S-phase. PC-3 cells present a functional mitotic spindle checkpoint [48] that requires a functional nuclear lamina. Therefore, in MeCP2 ablated cells, where the lamins are knock down, progression into G_2_/M might be precluded [49].

The NE creates a barrier between the nucleoplasm and the cytoplasm and constitutes a central element of intracellular architecture. Furthermore, NE components are essential for proper cell proliferation and actively participate in mitotic progression, revealing a tight interplay between NE components and the mitotic machinery [23,50]. In this view, the marked decrease in cell proliferation and changes in cell cycle progression observed following MeCP2 ablation could be explained by anomalous expression of NE components, with a consequent defect in the architecture of the nuclear envelope, rather than by an increase in apoptosis and/or senescence, phenotypes which might take over later. Although MeCP2 is largely known as a transcriptional repressor [51], KD of this protein in our experiments determines a decrease in levels of NE components, suggesting that MeCP2 might act either indirectly, for example by repressing transcriptional-repressor protein(s) of NE target genes, or directly as a transcriptional activator as previously suggested [52].

Lamin-depleted envelopes are extremely fragile and fail to grow beyond a limited extent highlighting that lamins play an essential role in later growth and in maintaining the structural integrity of the envelope. In fact, at the end of mitosis nuclear assembly occurs firstly by formation of an envelope capable of regulating nuclear-cytoplasmic transport and then the assembly of the lamina completes this initial envelope. During interphase, the lamina plays an essential role in maintaining the shape, volume and stability of the NE [49,53].

Dynamic reorganization of the NE is also essential for chromosome segregation during mitosis and restoration of the nuclear compartment in the daughter cells. During mid-late S-phase, B-type lamins are associated with the elongation phase of DNA replication, in agreement with the observation that B-type lamins, proliferating cell nuclear antigene (PCNA) and BrdU colocalise in nucleoplasmatic foci in cultured mammalian cells [54]. Moir and co-workers [55] also demonstrated that during the anaphase-telophase transition, lamin B1 begins to associate to the surface of the chromosomes. Finally, during the transition from late cytokinesis into G_1_ lamin B1 rapidly encloses the entire perimeter of the region containing decondensing chromosomes in each daughter cell. Therefore, the failure in cell proliferation following MeCP2 silencing could be explained by the decreased amount of lamins, which can contribute to generate defective NEs during S-, G_2_/M-phases and alterations in shape and resistance to mechanical stress of the NEs in the following cell cycle.

NE has scaffolding functions for the formation and regulation of higher order chromatin organization and in epigenetic regulatory pathways. Previous results point to the interaction between chromatin modifiers, epigenetic regulators, chromatin architectural organizers and NE components. In particular, the NET (Nuclear Envelope Transmembrane) protein LBR (Lamin B-binding Receptor) forms a quaternary complex with HP1 and H3/H4 histones [17] and binds to peripheral heterochromatin as a higher oligomer, thereby creating distinct nuclear envelope microdomains [56] involved in chromatin remodelling and transcriptional inactivation [57]. Besides, MeCP2 interacts with HP1 during differentiation within the heterochromatin regions [14] and, as already noted, MeCP2 binds LBR [18,19]. Thus, the results of this paper, together with data previously reported by our group [18], are consistent with a hypothesis that the deficiency of MeCP2 might cause the lack of a key “bridge” function that links the peripheral heterochromatin to the NE, accounting for a role of this protein in mediating the distribution of heterochromatin fractions at the nuclear periphery linked to the inner membrane.

## Conclusion

In summary, our results suggest that in absence of MeCP2 cells have reduced levels of NE components, such as lamins and LBR, thereby losing their ability to correctly assemble their NE, together with decreased in cell proliferation and viability. MeCP2 might be an important “bridge” protein between nuclear envelope and chromatin, providing chromatin stability at the nuclear periphery. MeCP2 could be involved in regulating the expression of NE components, ensuring that adequate levels are present to maintain proper NE organization. Further studies are necessary to better investigate, and to strengthen the evidence for this novel interesting role of MeCP2 and the intriguing link between this multifunctional protein and the architecture and organization of the NE.

## Methods

### Cell culture and transfection

PC-3 and LNCaP cells (ATCC) were cultured in RPMI 1640 (EuroClone) supplemented with 10% FCS (EuroClone) and 1X L-glutamine (PAA) and 1X Penicillin-Streptomycin (PAA). NIH-3 T3 cells (ATCC) were cultured in Dulbecco’s Eagle’s medium high glucose (DMEM) supplemented with 10% FCS (EuroClone) and 1X L-glutamine (PAA) and 1X Penicillin-Streptomycin (PAA).

Human or mouse MeCP2 RNA sequences (On-TARGETplus™ SMARTpool, Dharmacon RNA Technologies) and Non-targeting Pool (Dharmacon) were transfected with INTERFERin™ siRNA transfection reagent (Polyplus transfection) according to the manufacturer’s recommendations.

### Cell proliferation

PC-3, LNCaP and NIH-3 T3 cells were transfected with human/mouse MeCP2 SMARTpool (siRNA MeCP2) or Non-targeting Pool (siRNA CTRL) sequences. Six-wells plates were seeded with 4 × 10^4^ PC-3 cells per well, 8 × 10^4^ LNCaP cells per well and 3 × 10^4^ NIH-3 T3 cells and transfected the day after. All cell lines were retransfected at 3 and 5 days after the first transfection (total of three following transfections) and counted at 3, 5 and 7 days of silencing.

### Cell cycle analysis

For cell cycle analysis, PC-3 cells transfected (total time 7 days) were fixed in ice-cold 70% ethanol, washed in 1X PBS buffer, and treated with 20μg/ml RNaseA and 50 μg/ml Propidium Iodide (SIGMA-Aldrich St.Louis-MO) for 30 min at RT prior to analysis of 1 × 10^4^ cells with FACSCalibur flow cytometer (Becton-Dickinson, Franklin Lakes, NJ, USA). Data were processed using CellQuest software (Becton-Dickinson).

### Bi-parametric analysis

PC-3 cells were transfected for 7 days using human siRNA MeCP2 or human siRNA CTRL SMARTpool oligos, as described above. Cells were incubated with bromodeoxyuridine (BrdU; 10 μM for 30 minutes), harvested and fixed in ice-cold 70% ethanol. 1 × 10^6^ cells were exposed to 2 N HCl for 30 minutes at room temperature and, after centrifuge, to 0.1 M of Na_2_B_4_O_7_ 10H_2_O (pH = 8.5) for 10 minutes. After centrifuge, cells were resuspended in 0.5% Tween20/1% BSA/PBS 1X solution and incubated with anti-BrdU antibody (Becton Dickinson) at room temperature for 45 minutes. After centrifuge, cells were resuspended in 50 μl of 0.5% Tween20/1% BSA/PBS 1X solution added with anti-mouse FITC antibody (Alexa Fluor 488 F(ab’)_2_ fragment of goat anti-mouse IgG), 1:50 diluted, and incubated at room temperature for 30 minutes. Cells were centrifuged and resuspended in PBS 1X solution, treated with 20 μg/ml RNaseA and 5 μM Propidium Iodide (SIGMA-Aldrich St.Louis-MO) for 1 hour at RT, and analysed by FACSCalibur flow cytometer (Becton-Dickinson, Franklin Lakes, NJ, USA). Data were processed using CellQuest software (Becton-Dickinson). The distribution of green fluorescence from FITC, expressed on a logarithmic scale, was collected as a measure of BrdU incorporation, and the distribution of red fluorescence from PI, on a linear scale, was collected as a measure of DNA content.

### Annexin V assay (apoptosis detection)

PC-3 cells were silenced using human MeCP2 siRNA SMARTpool. Six-well plates were seeded with 4 × 10^4^ PC-3 cells per well and 24 hours after were silenced for 7 days (three subsequent transfections were performed, PC-3 were counted at 3, 5 and 7 days). At each counter passage, cells were prepared for cell cycle analysis (FACS preparation, see below) and Western blot assay (see below).

Apoptosis detection was performed using Annexin V-FITC Apoptosis Detection Kit (BD Pharmingen) by FACS assay.

### SA-β-galactosidase assay (cellular senescence detection)

PC-3 siMeCP2 transfected cells were tested for senescence using SA-β-galactosidase assay (Cellular Senescence assay Kit, Chemicon International-MILLIPORE) according to the manufacturer’s instructions.

### Western blot analysis and antibodies

Western blotting analyses were carried out using whole cell extracts obtained using Leammli buffer 2X, separated on 8–14% SDS-PAGE gels, and transferred to nitrocellulose membranes. Membranes were incubated with the following primary antibodies: anti-MeCP2 rabbit polyclonal (1:500 dilution, M 9317 SIGMA-Aldrich), anti-laminA/C goat polyclonal (1:500 dilution, sc-6215, SantaCruz Biotech., Santa Cruz, CA), anti-laminB1 goat polyclonal (1:500 dilution, c-20, Santa Cruz), anti-LBR rabbit polyclonal (1:2000 dilution, Covance Research Products INC), anti-Cleaved PARP (Asp214) rabbit polyclonal (1:1000 dilution, Cell Signaling Technology, Inc.). The corresponding peroxidase-labeled secondary antibody (Jackson ImmunoResearch, Laboratories, INC.) was detected using ECL western blotting reagents (sc-2048 Santa Cruz biotechnology, INC). Equal loading were checked out by GAPDH (anti-GAPDH rabbit polyclonal 1:2000 dilution, Open Biosystems).

### Immunofluorescence microscopy

PC-3 siRNA MeCP2 transfected cells were washed with 1X PBS buffer, fixed with 4% paraformaldehyde, permeabilized with 0.1% Triton X-100 (room temperature, 10 min) and blocked using 1X PBS buffer with 2% BSA, 30 min. In order to detect endogenous MeCP2 or lamin A proteins, cells were incubated with anti-MeCP2 rabbit polyclonal antibody (1:500 dilution, M9317 SIGMA) or with anti-lamin A mouse monoclonal antibody (1:500 dilution, Abcam Cambridge, UK) for 60 min. In order to detect senescent phenotype, cells were incubated with anti-HPγ mouse monoclonal antibody and anti-H3 (trimethylK9) rabbit polyclonal (both 1:100 dilution, Abcam Cambridge, UK). The coverslips were washed and incubated with secondary antibody conjugated to Cy™3-conjugated Affinity Donkey anti-rabbit antibody (1:500 dilution, Jackson ImmunoResearchLaboratories, INC) or Cy™2-conjugated Affinity Donkey anti-mouse antibody (1:500 dilution, Jackson) for 60 min. Cells were washed twice and incubated with DAPI, rinsed in 1X PBS. Finally, coverslips were washed and mounted with Moviol fluorescent mount. NE Images were acquired on a Zeiss LSM410 laser scanning confocal microscope using the 63X objective lens; senescent phenotype images were analysed with a fluorescence microscope (BX51; Olympus, Melville,NY; equipped with UplanIF 40X/0.75) and pictures were acquired with a colour camera (DP50; Olympus).

### RT-qPCR analysis

Primers to be used in RT-qPCR experiments were designed with on-line Primer3 software ( http://frodo.wi.mit.edu/) choosing amplicons of approximately 75-135 bp (see Additional file 1: Table S1). The selected sequences were tested against public databases (NBLAST) to confirm the identity of the genes.

PC-3 cell cultures were collected and lysed by addition of RP1 buffer (NucleoSpin TriPrep kit, Macherey-Nagel GMbH&Co.KG, Germany) and total RNA was isolated. To prevent genomic DNA contamination rDNaseI (RNase-free) was used according to the manufacturer’s instructions (NucleoSpin TriPrep kit, Macherey-Nagel GMbH&Co.KG, Germany). Quality of the total RNA was assessed using a Nanodrop (with A_260/280_ around 1,9-2,00) and on gel. 2 μg of total RNA were reverse-transcribed in 20 μl using SuperScript III First-Strand (Invitrogen) with oligo-dT primer, according to manufacturer’s instructions. cDNA was diluted 1:10 and subsequent real-time PCR reactions were carried out on a PTC-200 cycler (MJ Research). Real-time PCR were performed with 1X iQ™SYBR Green Supermix (BioRad) and 100nM primer forward and 100nM primer reverse final concentrations. In brief, the qPCR mixtures were pre-heated at 95°C for 10 min, followed by 40 cycles of amplification (95°C for 15 s, 58°C for 1 min, 80°C for 30 s) and melting curves analyses (from 60.5°C to 90°C, read every 0.5°C, hold 5 s). Blank (No Template Controls) and noRT controls were run for detecting PCR and DNA contamination, respectively. Data were analysed by averaging quadruplicates C_q_. Levels of RNA expression were determined by Gene Expression Analysis for iCycler iQ Real-Time PCR Detection System v1.10 (Bio-Rad) according to the 2-ΔΔC_q_ method. Levels of RNA expression of selected genes were normalised to the internal control GAPDH.

### Statistical analysis

Statistical analyses of the data were performed by two-way ANOVA, with Bonferroni’s post-test for multiple comparisons, or Student’s *t* test, using Prism 4.03 (GraphPad Software, Inc., San Diego, CA, USA).

## Competing interests

The authors declare that they have no competing interests.

## Authors’ contributions

FB performed the apoptosis detection assays, cellular senescence assays, immunofluorescences, RT-qPCR analyses and has contributed to the drafting of the manuscript. IC and CC performed cell cultures transfections and cell growth curves. MBG performed FACS analyses and statistical analyses. CP and EM performed western blot analyses. EM supervised the FACS analyses and edited the manuscript. GB supervised confocal microscopy and edited the manuscript. IMB conceived the study, participated in its design and coordination, and co-wrote the manuscript. All authors read and approved the final manuscript.

## Supplementary Material

Additional file 1 **Table S1.** Table of qPCR primers used. Click here for file
