# Supplementary material for: Knock-down of methyl CpG-binding protein 2 (MeCP2) causes alterations in cell proliferation and nuclear lamins expression in mammalian cells
Source: BMC Cell Biol. 2012 Jul 11;13:19. doi: 10.1186/1471-2121-13-19 (PMC3477090; doi:10.1186/1471-2121-13-19)
Supplement: Additional file 1 — Table S1. Table of qPCR primers used. [file 1471-2121-13-19-S1.pdf]

Additional file 1

Table 1S: qPCR primers

Target	Accession Number (NM_)	Position	Forward primer 5'→3'<br>Reverse primer 5'→3'	Tm °C	Amplicon size (bp)
MeCP2	001110792.1	983	F: GATCTGTGCAGGAGACCGTA<br>R: ACTTCCTTGACCTCGATGCT	60	76
LMNA (LaminA+C)	005572	1585	F: GATGAGGAGGGCAAGTTTGT<br>R: CCTTCAGGGTGAACTTTGGT	60	136
LMNA (Lamin A)	170707.2	2854	F: GTGAGTCCATTCTCCCAGGT<br>R: CTAGGGAAGGCAGCTCAAAC	60	124
LMNB1	001198557.1	233	F: AGGATCAGATTGCCCAGTTG<br>R: GCGAAACTCCAAGTCCTCAG	60	125
LBR	002296.3	763	F: TTTGAAGTGACCCCCATCC<br>R: GGCCAAACATGATGAGAAACA	60	76
GAPDH	002046	556	F: TGCACCACCAACTGCTTAGC<br>R: GGCATGGACTGTGGTCATGA	60	87
